# Halogen and Hydrogen Bonding in Halogenabenzene/NH_3_ Complexes Compared Using Next-Generation QTAIM

**DOI:** 10.3390/molecules24162875

**Published:** 2019-08-08

**Authors:** Shuman Li, Tianlv Xu, Tanja van Mourik, Herbert Früchtl, Steven R. Kirk, Samantha Jenkins

**Affiliations:** 1 Key Laboratory of Chemical Biology and Traditional Chinese Medicine Research and Key Laboratory of Resource; National and Local Joint Engineering Laboratory for New Petro-chemical Materials and Fine Utilization of Resources, College of Chemistry and Chemical Engineering, Hunan Normal, Changsha 410081, Hunan, China; 2 EaStCHEM School of Chemistry, University of St Andrews, North Haugh, St Andrews, Fife KY16 9ST, Scotland, UK

**Keywords:** halogen bonding, next-generation QTAIM, ZORA, DFT, double-hybrid density functional theory, halogenabenzene, halouracil

## Abstract

Next-generation quantum theory of atoms in molecules (QTAIM) was used to investigate the competition between hydrogen bonding and halogen bonding for the recently proposed (Y = Br, I, At)/halogenabenzene/NH_3_ complex. Differences between using the SR-ZORA Hamiltonian and effective core potentials (ECPs) to account for relativistic effects with increased atomic mass demonstrated that next-generation QTAIM is a much more responsive tool than conventional QTAIM. Subtle details of the competition between halogen bonding and hydrogen bonding were observed, indicating a mixed chemical character shown in the 3-D paths constructed from the bond-path framework set B. In addition, the use of SR-ZORA reduced or entirely removed spurious features of B on the site of the halogen atoms.

## 1. Introduction

Halogen bonding is the most widely studied of the collective σ-hole interactions. These interactions involve interaction of a nucleophile with a σ-hole, which is a region of depleted electron density [1]. In halogen bonds, the σ-hole is generally located at the extension of the A–X bond, where X is the halogen and A the atom it is covalently bonded to, usually carbon. As such, halogen bonds have a propensity to be linear, though significant deviations from linearity can occur if secondary interactions are present [2]. Although they are often weaker than hydrogen bonds, halogen bonds involving the heavier halogens, where the halogen acts as the electrophile, can be of similar or larger strength than corresponding hydrogen bonds with the halogen acting as the nucleophile; this is because the halogen bond strength increases with increasing size of the halogen, whereas the hydrogen bond strength does not. The dual character of halogens, which can act both as nucleophiles in hydrogen bonds and as electrophiles in halogen bonds, suggests there may be competition between halogen and hydrogen bonding when a halogenated molecule interacts with a ligand that can also act as both nucleophile or electrophile, such as NH_3_. Usually, the halogen is bonded to one atom, often a carbon, such as in the halogenated nucleic acid bases. One of the authors previously investigated the competition between halogen and hydrogen bonding in complexes of (X = F, Cl, Br, I, At)/1-methyluracil/H_2_O*_n_* (*n* = 1, 2) using density functional theory (DFT) [2]. It was found that structures with two waters forming a bridge between the C4=O and C5–X functional groups, through a combination of hydrogen and halogen bonding interactions, exist for all halogens except F, whereas no singly hydrated chlorinated 1-methyluracil complex exists. Thus, the absence of a halogen-bonded structure for chlorinated 1-methyluracil is attributed to the competing hydrogen bonding interaction. Recently, one of the authors investigated the ability of halogenabenzene—C_5_H_5_Y, where the halogen Y is bonded to two carbon atoms—to form halogen bonds [3]. Earlier, Rawashdeh et al. proposed, based on quantum chemical calculations, that the halogenabenzene structure adopts a non-planar C_s_-symmetric graceful “flying bird”-like structure [4]. Cates and Van Mourik showed that two positively charged σ-holes exist in the bird structure, roughly located at the extensions of the two C–Y bonds. The authors investigated the (Y = Br, I, At)/halogenabenzene/NH_3_ system [3] and concluded that a halogen bond and a hydrogen bond form with the NH_3_ nucleophile. We previously observed for the (X = F, Cl, Br, I, At)/1-methyluracil/H_2_O system that the strength of the hydrogen bond decreased with increasing halogen size, and the hydrogen bonds displayed an admixture of covalent character, but all the halogen bonds were purely electrostatic in nature [5].

Research into halogen-bonded systems has increased substantially over the last decade, and the literature contains a plethora of experimental and computational studies on halogen-bonded systems in different environments (e.g., gas phase, solution [6], solid state [7], and in clathrate cages [8]). The appearance of review articles [1,9,10,11,12,13,14] in the literature indicates the maturity of this field. For gas-phase systems, one of the most accurate methods is CCSD(T) (coupled cluster with single, double, and perturbative triple excitations). Halogen bonds have been studied via a wide range of computational methods. Kozuch and Martin built two benchmark sets for halogen-bonded systems, XB18 and XB51 [15]. They found that DFT methods with high exact exchange and long-range corrections were suitable, as well as double-hybrid functionals. 

Because the halogen complexes comprise heavier atoms, relativistic effects will be non-negligible [16]. Earlier, some of the authors of the current work demonstrated the superior performance of the scalar-relativistic zeroth-order regular approximation to the Dirac equation (SR-ZORA) [17] over the use of effective core potentials (ECPs) for a quantum theory of atoms in molecules (QTAIM) [18] bond critical point (*BCP*) investigation of the [Sb_3_Au_3_Sb_3_]^3−^ sandwich complex [19]. 

In this work we determined the extent to which the size of the halogen atom and the *BCP* and 3-D bond-path properties, i.e., next-generation QTAIM [20], are affected by the choice of using either pseudopotential relativistic calculations or SR-ZORA.

## 2. Theoretical Background

We used QTAIM [18] and stress tensor analysis that utilizes higher derivatives of the total charge density distribution *ρ*(**r_b_**) at the bond critical point (*BCP*), where the subscript “**_b_**” refers to the *BCP*. QTAIM analysis utilizes the gradient vector field ∇*ρ*(**r**) and provides four types of topologically stable critical points according to the set of ordered eigenvalues λ_1_ < λ_2_ < λ_3_, with corresponding eigenvectors **e_1_**, **e_2_**, and **e_3_** of the Hessian matrix. The complete set of critical points, together with the bond-paths, of a molecule or cluster is referred to as the molecular graph, with the constituent atoms being referred to as nuclear critical points (*NCP*s). The ellipticity ε provides the relative accumulation of *ρ*(**r_b_**) in the two directions perpendicular to the bond-path at a *BCP*, defined as ε = |λ_1_|/|λ_2_| **–** 1 where λ_1_ and λ_2_ are negative eigenvalues of the corresponding **e_1_** and **e_2_** eigenvectors, respectively. Previously, we defined bond-path *BCP* stiffness, S = |λ_2_|/λ_3_, as a measure of rigidity of the bond-path [21]. It has been shown [22,23] that the degree of covalent character can be determined from the total local energy density *H*(**r**_b_), defined as

*H*(**r**_b_) = *G*(**r**_b_) + *V*(**r**_b_) (1)

where *G*(**r**_b_) and *V*(**r**_b_) are the local kinetic and potential energy densities at a *BCP*, respectively. A value of *H*(**r**_b_) < 0 for the closed-shell interaction, ∇**^2^***ρ*(**r**_b_) > 0, indicates a *BCP* with a degree of covalent character; conversely, *H*(**r**_b_) > 0 reveals a lack of covalent character for the closed-shell *BCP*. In the terminology used throughout this work, “--" refers to closed-shell *BCP*s which by definition always possess values of the Laplacian ∇^2^*ρ*(**r**_b_) > 0, but can possess *H*(**r**_b_) < 0 or *H*(**r**_b_) > 0. Conversely, “-“ always refers to shared-shell *BCP*s which by definition always possess values of the Laplacian ∇^2^*ρ*(**r**_b_) < 0 and *H*(**r**_b_) < 0. A related quantity to the ellipticity ε for closed-shell interactions is the metallicity:

ξ(**r**_b_) = *ρ*(**r**_b_)/∇^2^*ρ*(**r**_b_) ≥ 1 (2)

where the values of total electronic charge density *ρ*(**r**_b_) and the Laplacian ∇^2^*ρ*(**r**_b_) are calculated at the *BCP*. The metallicity ξ(**r**_b_) [24,25] has previously been used to explore the suspected metallicity ranges of metals, metalloids, and non-metals [24,25] and has been demonstrated to be inversely related to the “nearsightedness” of the first-order density matrix and to be suitable for closed-shell systems [26]. 

The bond-path length (BPL) is defined as the length of the path traced out by the **e_3_** eigenvector of the Hessian of the total charge density *ρ*(**r**), passing through the *BCP*, along which *ρ*(**r**) is locally maximal with respect to any neighboring paths. The bond-path curvature is a dimensionless ratio separating two bonded nuclei and is defined as

(BPL – GBL)/GBL(3)

where the geometric bond length (GBL) refers to the inter-nuclear separation. The BPL can exceed the GBL for weak or strained bonds existing in unusual bonding environments [27]. For 3-D bond-paths, there are minor and major radii of bonding curvature, specified by the directions of **e_2_** and **e_1_**, respectively [28]. 

We refer to the next-generation QTAIM interpretation of the chemical bond as the bond-path framework set, denoted by B, where B = {***p*,*q*,*r***}, with the consequence that for a given electronic state a bond comprises three “linkages”: ***p***, ***q***, and ***r***, associated with the **e_1_**, **e_2_**, and **e_3_** eigenvectors, respectively. Here, ***p*** and ***q*** are 3-D paths constructed from the values of the least (**e_1_**) and most (**e_2_**) preferred directions of electronic charge density accumulation *ρ*(**r**) along the bond-path, referred to as (***r***). An in-depth discussion with derivations of B = {***p*,*q*,*r***} is provided in the Supplementary Materials S1.

The orbital-like *packet* shapes that the pair of ***q***- and ***q***’-paths form along the *BCP* are referred to as a {***q***,***q*’**} path-packet. Extremely long {***q****,**q’***}-paths indicate imminent rupture caused by the coalescence of a *BCP* with the associated *RCP* (ring critical point). Larger {***q,q’***} path-packets in the vicinity of a *BCP* signify an easier passage of the *BCP* and, hence, of the associated *NCP*, as opposed to smaller {***q,q’***} path-packets. The lengths of the {***q***,***q*’**} and {***p***,***p*’**} path-packets are calculated as (H,H’) and (H^*^,H^*^’), respectively, using the ellipticity ε as the scaling factor (see the Supplementary Materials S1).

In this investigation, we also considered B_σ_ = {***p***_σ_**,*q***_σ_**,*r***} using Bader’s definition [29,30] of the quantum stress tensor σ(**r**) that has provided a physical explanation of the low-frequency normal modes that accompany structural rearrangement [31,32,33] and is directly related to the Ehrenfest force by the virial theorem.

## 3. Computational Details

To obtain the wavefunctions using ECPs, the complexes of NH_3_ interacting with the halogenabenzene structures with Y = Cl, Br, I, and At were optimized using the mPW2-PLYP double-hybrid density functional [34] and the aug-cc-pVTZ basis set [35,36] for all atoms except Y = I, At. For the I and At atoms, the aug-cc-pVTZ-PP basis set [37,38] was used, which includes relativistic ECPs. Double-hybrid functionals have been identified as the most accurate density functionals for ground-state thermochemistry [39]. A method comparison focal study on iodobenzene•••H_2_O in previous work [3] showed that mPW2-PLYP/aug-cc-pVTZ(-PP) gives interaction energies and halogen bond distances in excellent agreement with DLPNO-CCSD(T)/ma-def2-QZVP results, evidencing that this level of theory is accurate for studying halogen bonds. All geometry optimizations were performed with Gaussian09 Rev. E.01 [40] and used Gaussian’s “ultrafine” integration grid and “very tight” (RMS force < 10^−6^ Hartree/au) convergence criteria. 

For the wavefunctions with the SR-ZORA approximation for Y = F, Cl, Br, single-point calculations were performed on the optimized structures using the ORCA code [41] using mPW2-PLYP [34], the built-in ZORA form of the ma-ZORA-def2-TZVP basis set [42], very tight convergence parameters, and a fine integration grid (GRID7). For Y = I, At, the integration grid accuracy parameter was tightened to 10^−10^, the other simulation parameters remaining the same as for F, Cl, and Br. Additionally, for Y = I the “old-ZORA-TZVP” basis set was substituted for ma-ZORA-def2-TZVP specifically on the I atom, and for Y = At the “SARC-ZORA-TZVP” basis set [43,44] was substituted for ma-ZORA-def2-TZVP specifically on the At atom.

These calculations yielded the wave functions needed for QTAIM analysis; calculations of the molecular graphs and critical point properties were performed using AIMAll [45]. All molecular graphs were additionally confirmed to be free of non-nuclear attractor critical points. The calculated paths comprising B and B_σ_ were visualized using the Python 3 visualization toolkit Mayavi [46].

## 4. Results and Discussion

### 4.1. The QTAIM and Stress Tensor BCP and Bond-Path Properties of the (Y = Cl, Br, I, At)/NH_3_ System

In this section, firstly we investigated the competition between halogen bonding and hydrogen bonding for the (Y = Br, I, At)/halogenabenzene/NH_3_ and (X = F, Cl, Br, I, At) system. The original results are provided in Table 1 and Table 2 and Table S4 of the Supplementary Materials. Secondly, we considered the effect of an increase in atomic mass of the halogen atom on the *BCP* and bond-path properties. The lengths of the eigenvector following paths (H,H’) and (H^*^,H^*^’) of the {***q***,***q*’**} and {***p***,***p’***} path-packets corresponding to the halogen bond (present at ∆E_min_) Y--N12 *BCP*s of the (Y = Br, I, At)/halogenabenzene/NH_3_ system are competitive, on the basis of similarity, with those of the hydrogen bonds where they are present (see Table 1). The S values of the hydrogen bond Cl2--N14 *BCP* are much greater for the hydrogen bond Br2--N14 *BCP*, indicating that the Y = Cl system possesses the most stable hydrogen bond of the series. There is an increase in S values for the progression Y = Br, I, At, indicating an increase in topological stability of the Y--N12 *BCP* with increasing atomic mass of the halogen atom. 

There is a transition in the connectivity of the halogenabenzene/NH_3_ systems from the lightest through to the heaviest halogen atoms. For Y = Cl, there is only a hydrogen bond C3--H14 *BCP* and no halogen bond. Intermediate is Y = Br, where there is both the hydrogen bond C3--H14 *BCP and* the halogen bond Br2--N12 *BCP.* Finally, the heavier halogen atoms Y = I, At both contain a halogen bond, I2--N12 *BCP* and At6--N12 *BCP*, respectively, but no hydrogen bond *BCP*s. 

**Figure 1:**
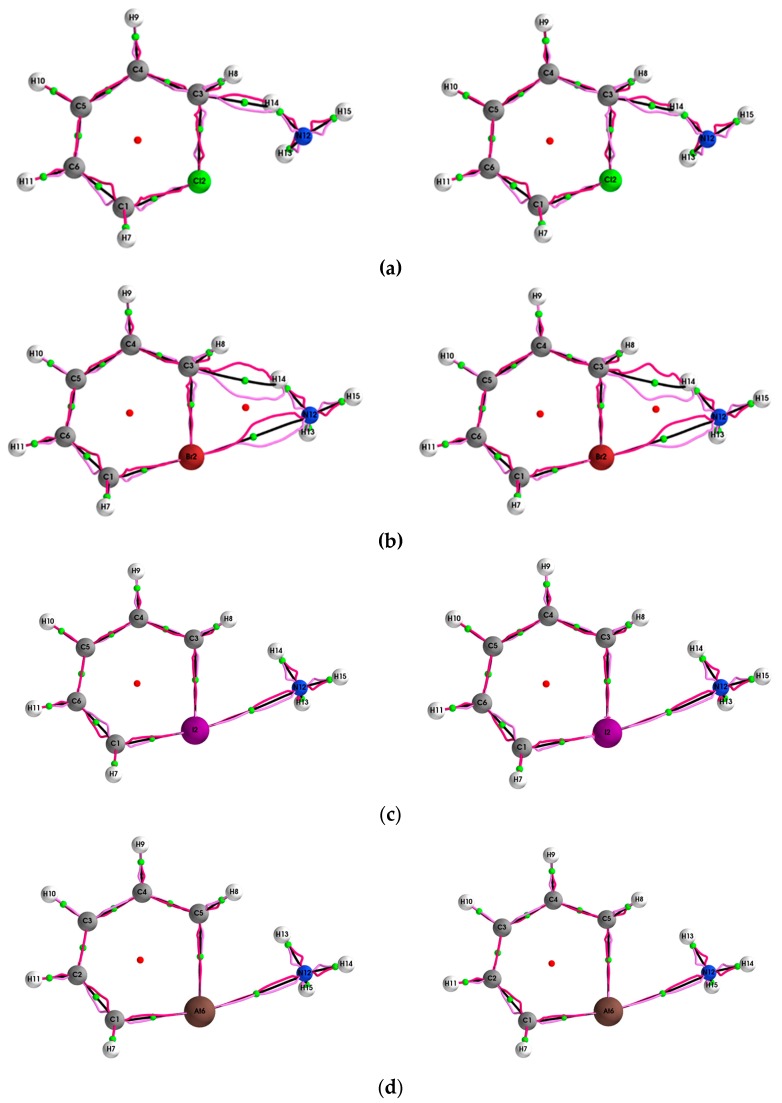
The **{*q,q’*}** path-packets; ***q*** (dark magenta) and ***q*****ʹ** (light magenta) for the (Y = Cl, Br, I, At)/halogenabenzene/NH_3_ system are presented in subfigures **(a–d)**, respectively, calculated using ECPs (left panels) and with the SR-ZORA Hamiltonian (right panels).

There is a dependency on atomic mass of the halogen atom apparent in the variation of the lengths of the {***q***,***q*’**} and {***p***,***p’***} path-packets given by (H,H’) and (H*,H*’). The {***q***,***q*’**} and {***p***,***p’***} path-packets of the closed shell *BCP*s—the halogen bonds and hydrogen bonds with the greatest tendency to rupture, i.e., those with greater topological instability—are associated with longer BPL and paths (H,H’) and (H*,H*’). For the hydrogen bond C3--H14 *BCP*, the (H,H’) and (H*,H*’) values are lower for Y = Cl compared with Y = Br, demonstrating a smaller tendency towards the rupture of the hydrogen bond C3--H14 *BCP* for Y = Cl. For the halogen bond Y--N12 *BCP* for Y = Br, I, At, the (H,H’) and (H*,H*’) values are Br > I > At. Therefore, the order of decreasing tendency to rupture the halogen bonds is Br2--N12 *BCP* < I2--N12 *BCP* < At2--N12 *BCP*. In addition, for the halogen bonds, the {***q***,***q*’**} path-packets become more polarized towards the N12 *NCP* with increasing halogen atomic number from Y = Br, to I, to At. We note that the {***q***,***q*’**} path-packets envelop the Br2--N12 *BCP* but do not envelop the I2--N12 *BCP* and At2--N12 *BCP* (see Figure 1). Agreement was found with the stress tensor results in the form of the trends of the stress tensor (H_σ_, H_σ_’) and (H_σ_*,H_σ_*’) values. The tabulated results obtained with the ECPs are provided in Table S4 of the Supplementary Materials.

### 4.2. Comparison of SR-ZORA and ECPs for QTAIM and Stress Tensor Properties of the (Y = Cl, Br, I, At)/NH_3_ System

In this section, we compare the results corresponding to the difference ∆{(ECP) – (SR-ZORA)} to see the extent to which differences propagate away from the halogen *NCP* (atom) and any dependency on the atomic mass of the halogen atom (see Table 2).

**Figure 2:**
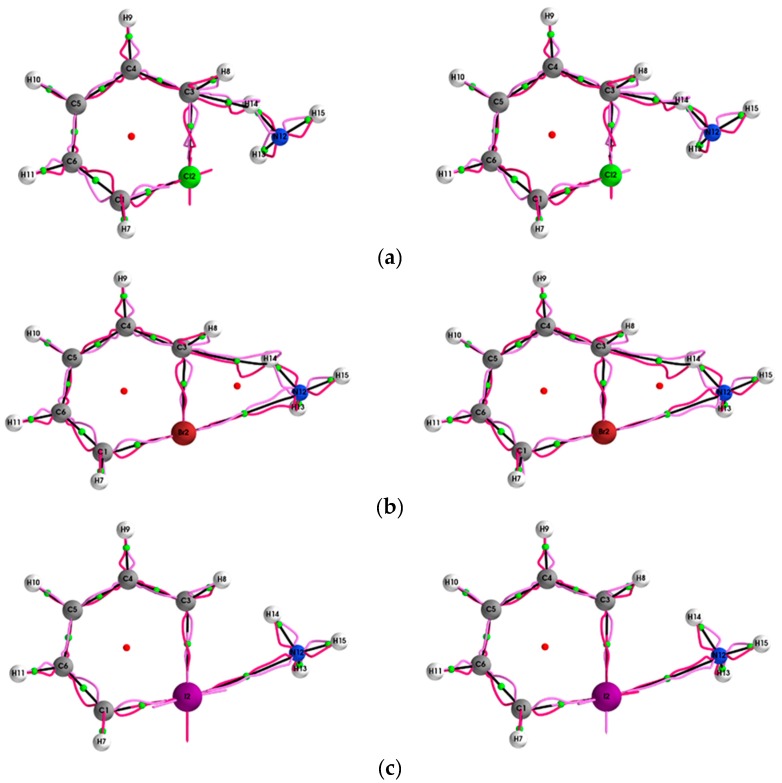

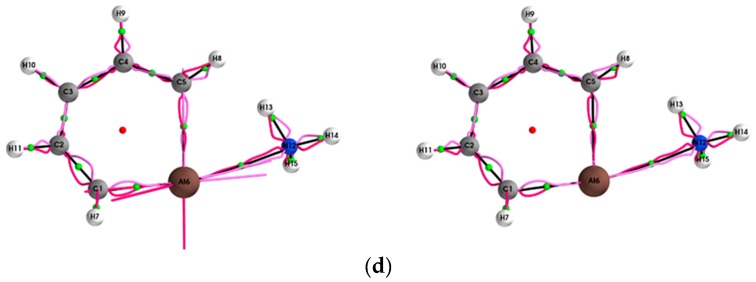
The {***q***_σ,_***q***_σ_**ʹ}** path-packets with a magnification factor of ×5 for the (Y = Cl, Br, I, At)/halogenabenzene/NH_3_ system are presented in subfigures (**a–d**), respectively; see the caption of Figure 1 for further details.

**Figure 3:**
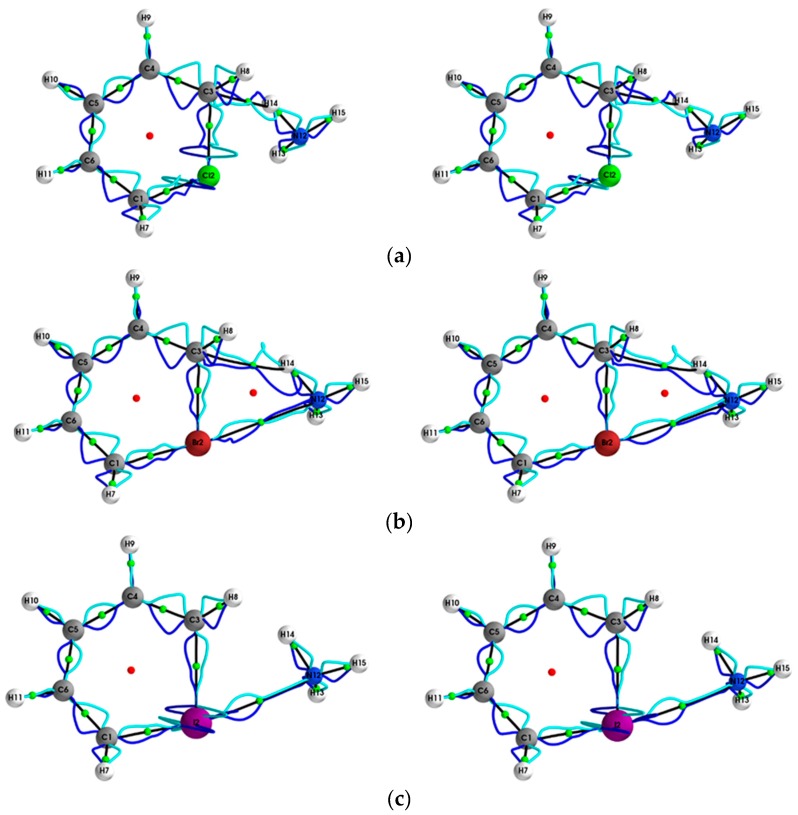

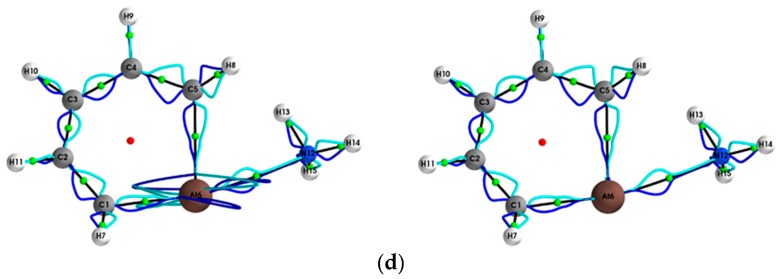
The {***p***_σ,_***p***_σ_**ʹ}** path-packets with a magnification factor of ×5 for the (Y = Cl, Br, I, At)/halogenabenzene/NH_3_ system are presented in subfigures **(a–d)**, respectively; see the caption of Figure 1 for further details.

The **{*q*,*q’*}** path-packets associated with the most preferred direction for the (Y = Cl, Br, I, At)/halogenabenzene/NH_3_ systems are presented both using ECPs (left panels) and SR-ZORA (right panels) in Figure 1. The corresponding results for the **{*p*,*p’*}** path-packets associated with the least preferred direction are provided in the Supplementary Materials S3. Similarly, for the stress tensor of the (Y = Cl, Br, I, At)/halogenabenzene/NH_3_ system, the **{*q***_σ_**,*q***_σ_***’*}** and **{*p***_σ_**,*p***_σ_***’*}** are presented in Figure 2 and Figure 3, respectively. 

There is a small effect on the conventional QTAIM property of the halogen bond BPL, which increases with halogen atom atomic mass; the SR-ZORA BPL is longer than the ECP BPL, evident from the sign of ∆BPL. The significance of the presence of a high degree of metallic character ξ(**r**_b_) ≥ 1 is that it indicates a high degree of delocalization of the electronic charge density associated with the bonding, i.e., that the bonding is highly electron deficient.

The effect on both the halogen bond and hydrogen bond path {***q***,***q*’**}, {***p***,***p’***} lengths ∆(H,H’), ∆(H*,H*’) and **{*q***_σ_**,*q***_σ_***’*}**, **{*p***_σ_**,*p***_σ_***’*}** lengths ∆(H_σ_,H_σ_’), ∆(H_σ_*,H_σ_*’) due to the use of SR-ZORA also increases with atomic mass; however, unlike for the BPL, the increase is significant, e.g., 2.5% for the At6--N16 *BCP* halogen bond. The variation of the properties calculated using QTAIM and the stress tensor is caused by the stress tensor being calculated within the QTAIM partitioning. In addition, very significant increases in the ∆(H,H’), ∆(H*,H*’) lengths and decreases in the ∆(H_σ_,H_σ_’), ∆(H_σ_*,H_σ_*’) lengths were found even for the strong shared-shell *BCP*s (see Table 2). The effect of SR-ZORA for the shared-shell *BCP*s also increases significantly with atomic mass. Inspection of the {***q***,***q*’**} path-packets of the hydrogen bond C3--N14 *BCP* for Y = Cl, Br, calculated with SR-ZORA (right panels) demonstrates the mix of chemical bond character evident as the double peaks of the {***q***,***q*’}**; this effect is more apparent for Y = Br. 

This mix of chemical character is not evident for the results obtained using ECPs (left panels) and is also observed for Y = Br for the stress tensor **{*q***_σ_**,*q***_σ_***’*}** (see Figure 2). SR-ZORA also decreased or entirely removed the spurious long straight lines on the Y = Cl, I, At *NCP*s for **{*q***_σ_**,*q***_σ_***’*}** and the spurious effects on the Y = Cl, I, At *NCP*s for **{*p***_σ_**,*p***_σ_***’*}** (see Figure 3).

## 5. Conclusions

In this investigation, we demonstrated that conventional QTAIM is entirely sufficient to assess the competition between halogen bonding and hydrogen bonding. Next-generation QTAIM, however, provides more subtle details, including the change in chemical character evident as the mixture of chemical character in the morphology of the {***q***,***q***’} path-packets. Next-generation QTAIM demonstrates that the use of ZORA is more advantageous than ECPs with respect to the detection of errors, quantified as the difference from the ZORA approximation, that increase with increasing atomic weight. In addition, the use of ECPs leads to more and larger spurious features. Conventional QTAIM is not sensitive enough to detect differences in the results from the use of ECPs compared with the ZORA approximation that scale with atomic number.

In particular, we firstly examined the competition of hydrogen bonding and halogen bonding as a function of increasing atomic mass of the halogen atoms that comprise the (Y = Cl, Br, I, At)/halogenabenzene/NH_3_ systems. Secondly, we investigated the effect of the use of the SR-ZORA Hamiltonian compared with standard effective core potentials (ECPs) to account for relativistic effects by comparing the {***q***,***q*’**}, {***p***,***p’***} and {***q***_σ_**,*q***_σ_*’*}, {***p***_σ_**,*p***_σ_***’***} path-packets and the tabulated differences of QTAIM and stress tensor *BCP* and bond-path properties in the form ∆{(ECP) − (SR-ZORA)}. Next-generation QTAIM was demonstrated to be a much more responsive measure for determining the effects of the inclusion of the SR-ZORA relativistic correction effects than the conventional QTAIM properties, such as the bond-path length (BPL) or *BCP* properties. 

The competition between halogen bonding and hydrogen bonding was seen to favor hydrogen bonding the most for the lightest halogen atom Y = Cl and halogen bonding for the heaviest halogens Y = I, At; intermediate behavior was observed for Y = Br, which contained both halogen and hydrogen bonding. For Y = Cl, there was no halogen bonding, and Y = I, At contained only halogen bonding. Whereas it is known that the halogen bond strength increases with the size of the halogen, our next-generation QTAIM investigation shed light on this phenomenon from a different perspective. 

The increasingly mixed chemical character of the hydrogen bonding present for Y = Cl, Br observed in the double peaks in the {***q***,***q*’**} path-packets was only evident with the use of SR-ZORA. This mix in chemical character demonstrates the competition between hydrogen bonding and halogen bonding, as the stronger double peak apparent for Y = Br indicates that the hydrogen bonding is more polarized and strained than the Y = Cl hydrogen bond.

The effects of the use of SR-ZORA on the calculated *BCP* and bond-path properties, for the halogen bonds, increased significantly with increasing atomic mass. The bond-path properties of the shared-shell *BCP*s were also strongly affected by the use of SR-ZORA. This demonstrates that the effects of SR-ZORA propagated along the entirety of the bond-path and were not only focused in the immediate vicinity of the halogen atoms (*NCP*s). Therefore, analysis of halogen bonding in future should include the use of the SR-ZORA approximation for regions beyond the immediate vicinity of the halogen atom, i.e., lone pairs and σ-holes, but also for bond-path and *BCP* properties.

## Figures and Tables

**Table 1:** SR-ZORA results for the eigenvector following path lengths (H,H’) and (H*,H*’), bond-path length (BPL), geometric bond length (GBL), (H_σ_,H_σ_’) and (H_σ_*,H_σ_*’), metallicity ξ(**r_b_**), stiffness S, and the total local energy density H(**r_b_**) in a.u. for the (Y = Cl, Br, I, At)/NH_3_ system. Note the use of the bond notations “--“ (closed-shell *BCP*) and “-“ (shared-shell *BCP*). The corresponding results obtained with effective core potentials (ECPs) are provided in Table S4 of the Supplementary Materials.

*BCP*	(H,H’)	(H^*^,H^*^’)	(BPL, GBL)	(H_σ_,H_σ_’)	(H_σ_^*^,H_σ_^*^’)	ξ(r_b_)	S	*H*(r_b_)
**Cl–NH_3_**								
C3--H14	(5.390, 5.202)	(5.266, 5.286)	(4.927, 4.864)	(5.024, 4.928)	(4.968, 4.978)	0.351	0.156	0.001
N12-H14	(2.161, 2.154)	(2.158, 2.158)	(1.877, 1.914)	(1.897, 1.897)	(1.897, 1.897)	−0.198	1.496	−0.481
C3-H8	(2.317, 2.298)	(2.294, 2.321)	(2.007, 2.036)	(2.051, 2.039)	(2.041, 2.049)	−0.274	1.426	−0.299
C4-C3	(3.105, 3.095)	(3.082, 3.119)	(2.586, 2.585)	(2.648, 2.631)	(2.637, 2.642)	−0.324	1.931	−0.375
Cl2-C3	(3.876, 3.831)	(3.839, 3.860)	(3.421. 3.396)	(3.999, 3.983)	(3.859, 3.905)	−0.862	0.776	−0.123
**Br–NH_3_**								
C3--H14	(6.297, 5.619)	(5.870, 5.905)	(5.125, 4.991)	(5.347, 5.097)	(5.212, 5.223)	0.329	0.115	0.001
Br2--N12	(6.429, 6.376)	(6.358, 6.437)	(6.107, 6.091)	(6.137, 6.133)	(6.129, 6.139)	0.286	0.108	0.001
N12-H14	(2.157, 2.149)	(2.153, 2.153)	(1.876, 1.913)	(1.896, 1.896)	(1.896, 1.896)	−0.198	1.500	−0.482
C3-H8	(2.294, 2.275)	(2.271, 2.298)	(2.008, 2.037)	(2.051, 2.038)	(2.040, 2.048)	−0.276	1.427	−0.297
C4-C3	(3.062, 3.054)	(3.038, 3.079)	(2.587, 2.585)	(2.646, 2.627)	(2.634, 2.639)	−0.325	1.927	−0.374
Br2-C3	(3.895, 3.859)	(3.871, 3.879)	(3.691, 3.666)	(3.716, 3.713)	(3.705, 3.724)	−1.103	0.717	−0.088
**I–NH_3_**								
I2--N12	(6.213, 6.195)	(6.198, 6.202)	(5.986, 5.970)	(6.588, 6.584)	(6.495, 6.564)	0.346	0.153	0.001
C3-H8	(2.250, 2.231)	(2.229, 2.252)	(2.010, 2.039)	(2.045, 2.035)	(2.036, 2.044)	−0.279	1.430	−0.295
C4-C3	(3.007, 3.002)	(2.985, 3.026)	(2.589, 2.587)	(2.641, 2.624)	(2.632, 2.634)	−0.329	1.905	−0.369
I2-C3	(4.363, 4.334)	(4.337, 4.343)	(4.025, 4.004)	(4.640, 4.638)	(4.542, 4.628)	−2.200	0.600	−0.061
**At–NH_3_**								
At6--N12	(6.160, 6.142)	(6.143, 6.146)	(5.927, 5.914)	(5.948, 5.943)	(5.939, 5.944)	0.357	0.158	0.001
C5-H8	(2.257, 2.238)	(2.237, 2.259)	(2.014, 2.043)	(2.051, 2.041)	(2.042, 2.050)	−0.280	1.429	−0.292
C4-C5	(2.963, 2.957)	(2.939, 2.982)	(2.593, 2.591)	(2.644, 2.626)	(2.633, 2.636)	−0.329	1.910	−0.367
At6-C5	(4.636, 4.616)	(4.622, 4.623)	(4.235, 4.208)	(4.272, 4.270)	(4.248, 4.265)	−22.03	0.486	−0.046

**Table 2:** The percentage difference of ∆{[(ECP) – (SR-ZORA)]/(ECP)} (%) for ∆(H,H’), ∆(H*,H*’), ∆(BPL), ∆(GBL), ∆(H_σ_,H_σ_’), ∆(H_σ_*,H_σ_*’), ∆ξ(**r_b_**), ∆S, and ∆*H*(**r_b_**) in a.u. of the (X = Cl, Br, I, At)/1-methyluracil/NH_3_ system; see Table S4 of the Supplementary Materials and Table 1 for the original ECP and SR-ZORA results, respectively, and the caption of Table 1 for further details.

*BCP*	∆(H,H’) (%)	∆(H*,H*’) (%)	∆(BPL, GBL) (%)	∆(H_σ_,H_σ_’) (%)	∆(H_σ_*,H_σ_*’) (%)	∆ξ(r_b_) (%)	∆S (%)	∆*H*(r_b_) (%)
**Cl–NH_3_**								
C3--H14	(−0.242, 0.115)	(−0.171, −0.019)	(−0.102, 0.000)	(−0.259, 0.000)	(−0.202, −0.101)	−3.540	−0.645	0.000
N12-H14	(0.231, 0.185)	(0.185, 0.185)	(−0.321, 0.000)	(−0.264, −0.264)	(−0.264, −0.264)	−5.319	8.502	8.031
C3-H8	(2.030, 1.921)	(1.966, 2.026)	(−0.200, 0.000)	(0.195, 0.049)	(0.098, 0.098)	−11.38	18.05	12.57
C4-C3	(2.908, 2.826)	(2.868, 2.895)	(0.039, 0.000)	(0.339, 0.303)	(0.265, 0.339)	−13.68	36.40	10.29
Cl2-C3	(3.003, 2.643)	(2.786, 2.746)	(0.175, 0.000)	(1.502, 1.872)	(1.656, 1.563)	−15.70	9.240	6.107
**Br–NH_3_**								
C3--H14	(−0.671, −0.807)	(−1.224, −0.854)	(−0.039, 0.000)	(−0.300, 0.157)	(−0.192, −0.173)	−1.543	−2.679	0.000
Br2--N12	(−0.047, 0.530)	(0.235, 0.294)	(0.000, 0.000)	(0.114, −0.114)	(−0.147, 0.065)	−4.380	−5.882	50.00
N12-H14	(0.416, 0.371)	(0.370, 0.416)	(−0.267, 0.000)	(−0.264, −0.264)	(−0.264, −0.264)	−5.319	8.369	8.015
C3-H8	(1.587, 1.515)	(1.518, 1.627)	(−0.150, 0.000)	(0.097, 0.000)	(0.049, 0.049)	−11.74	18.32	12.90
C4-C3	(2.608, 2.553)	(2.597, 2.594)	(0.000, 0.000)	(0.264, 0.266)	(0.265, 0.265)	−13.64	36.34	10.31
Br2-C3	(2.209, 1.906)	(2.050, 1.946)	(0.135, 0.000)	(0.215, 0.242)	(0.135, 0.321)	−7.926	2.846	1.124
**I–NH_3_**								
I2--N12	(−2.339, −2.295)	(−2.243, −2.276)	(−0.050, 0.000)	(0.768, 0.813)	(−0.480, 1.515)	−2.065	−2.000	0.000
C3-H8	(−0.852, −0.768)	(−0.814, −0.806)	(−0.100, 0.000)	(−0.196, −0.197)	(−0.197, −0.245)	−10.28	15.48	11.68
C4-C3	(−1.008, −0.975)	(−0.981, −1.035)	(0.000, 0.000)	(−0.152, −0.114)	(−0.152, −0.152)	−10.77	28.89	8.889
I2-C3	(−4.880, −4.965)	(−4.683, −4.827)	(−0.025, 0.000)	(1.003, 1.003)	(−0.643, 1.845)	−19.24	3.692	4.688
**At–NH_3_**								
At6--N12	(−2.564, −2.555)	(−2.452, −2.467)	(−0.034, 0.000)	(18.07, 18.13)	(12.62, 19.96)	−4.386	−1.282	0.000
C5-H8	(−0.267, −0.314)	(−0.314, −0.311)	(−0.099, 0.000)	(−0.146, −0.196)	(−0.196, −0.196)	−10.24	15.29	11.78
C4-C5	(−0.407, −0.373)	(−0.376, −0.404)	(0.000, 0.000)	(−0.114, −0.114)	(−0.076, −0.114)	−10.40	27.92	8.706
At6-C5	(−7.042, −7.174)	(−7.040, −7.039)	(0.000, 0.000)	(23.23, 23.24)	(17.11, 25.71)	1.432	−0.621	2.128

## Supplementary Materials

The following are available online. The research data underpinning this publication can be accessed at https://doi.org/10.17630/77e6b08a-74b4-466a-ba27-4a62a1927089.
