# Supplementary material for: Halogen and Hydrogen Bonding in Halogenabenzene/NH3 Complexes Compared Using Next-Generation QTAIM
Source: Molecules. 2019 Aug 8;24(16):2875. doi: 10.3390/molecules24162875 (PMC6720212; doi:10.3390/molecules24162875)
Supplement: Supplementary file 1 [file molecules-24-02875-s001.pdf]

SUPPLEMENTARY MATERIALS

Halogen and Hydrogen Bonding in Halogenabenzene/NH_3 Complexes Compared Using Next-Generation QTAIM

Shuman Li, Tianlv Xu [1], Tanja van Mourik [2,*], Herbert Früchtl [2], Steven R. Kirk [1,*] and Samantha Jenkins [1,*]

[1] Key Laboratory of Chemical Biology and Traditional Chinese Medicine Research and Key Laboratory of Resource; National and Local Joint Engineering Laboratory for New Petro-chemical Materials and Fine Utilization of Resources, College of Chemistry and Chemical Engineering, Hunan Normal, Changsha, Hunan 410081, China;

[2] EaStCHEM School of Chemistry, University of St Andrews, North Haugh, St Andrews, Fife KY16 9ST, Scotland, United Kingdom;

* Correspondence: tanja.vanmourik@st-andrews.ac.uk (T.v.M.); samanthajsuman@gmail.com (S.J.); steven.kirk@cantab.net (S.R.K.).

1. Supplementary Materials S1. A discussion of the construction of the bond-path framework set B.

2. Supplementary Materials S2. Outline of the ZORA Hamiltonian methodology.

3. Supplementary Materials S3. The {*p*,*p′*} path-packets for the (Y = Cl, Br, I, At): halogenabenzene: NH_3 system with ECPs.

4. Supplementary Materials S4. Table of Next Generation QTAIM results obtained with ECPs for the (Y = Cl, Br, I, At): 1-methyluracil: NH_3 system.

1. Supplementary Materials S1. A discussion of the construction of the bond-path framework set B

The reasons for the choice of the ellipticity ε as scaling factor. This was motivated by the fact that the scaled vector tip paths drop smoothly onto the bond-path, ensuring that the tip paths are always continuous. We previously discussed in detail the unsuitability of alternative scaling factors that included $|\lambda_1 - \lambda_2|$ but this and other choices were not used because they lack the universal chemical interpretation of the ellipticity ε e.g. double-bond ε > 0.25 vs. single bond character ε ≈ 0.10. Also unsuitable choices for scaling factors, on the basis of not attaining zero, included either ratios involving the λ_1 and λ_2 eigenvalue or any inclusion of the λ_3 eigenvalue. The λ_3 eigenvalue was also found to unsuitable because it contains no information about the least ($\underline{\mathbf{e}}_1$) and most ($\underline{\mathbf{e}}_2$) preferred directions of the total charge density $\rho(\mathbf{r})$ accumulation.

Discussion on the uniqueness of the H* **and** H. Because the scaling factor, ε_i is identical in equation **(3a)** and equation **(3b)** and H* and H are defined by the distances swept out by the $\underline{\mathbf{e}}_2$ tip path points $\boldsymbol{p}_i = \boldsymbol{r}_i + \varepsilon_i\underline{\mathbf{e}}_{1,i}$ and $\boldsymbol{q}_i = \boldsymbol{r}_i + \varepsilon_i\underline{\mathbf{e}}_{2,i}$ respectively then H* = H will result in a linear bond-path ***r*.** The bond-path framework set B = {***p***,***q***,***r***} should consider the bond-path to comprise the *unique* ***p***-, ***q***- and ***r***-paths, swept out by the $\underline{\mathbf{e}}_1$, $\underline{\mathbf{e}}_2$ and $\underline{\mathbf{e}}_3$, eigenvectors that form the eigenvector-following paths with lengths H*, H and BPL respectively. The ***p***- and ***q***-paths are unique even when the lengths of H* and H are the same or very similar because the ***p***- and ***q***-paths traverse different regions of space. Bond-paths ***r*** with non-zero bond-path curvature are more likely to occur for the equilibrium geometries of closed-shell *BCP*s than for shared-shell *BCP*s and will result in H* and H with different values. This is because the ***p***- and ***q***-paths will be different because of the greater distance travelled around the outside of a twisted bond-path ***r*** compared with the inside of the same twisted bond-path ***r***. This is because within QTAIM the $\underline{\mathbf{e}}_1$, $\underline{\mathbf{e}}_2$ and $\underline{\mathbf{e}}_3$, eigenvectors can only be defined to within to a factor of -1, i.e. ($\underline{\mathbf{e}}_1$,-$\underline{\mathbf{e}}_1$), ($\underline{\mathbf{e}}_2$,-$\underline{\mathbf{e}}_2$) and ($\underline{\mathbf{e}}_3$,-$\underline{\mathbf{e}}_3$) therefore there will be two possible tip-paths. The consequences of this (within QTAIM) calculation of the H* is that we dynamically update the sign convention to define H* as being the shorter of the two possible tip-paths because $\underline{\mathbf{e}}_1$ is the least preferred direction of accumulation of $\rho(\mathbf{r})$. A similar procedure is used for H except that we chose the longer of the two possible tip-paths because $\underline{\mathbf{e}}_2$ is the most preferred direction of accumulation of $\rho(\mathbf{r})$.

It should be noted that the direction of the ***p***- and ***q***-paths always remain orthogonal to each other since they are constructed from the $\underline{\mathbf{e}}_1$ and $\underline{\mathbf{e}}_2$ eigenvectors respectively. The ellipticity ε is used as a scaling factor in the construction of the ***p***- and ***q***-paths:

$$\boldsymbol{p}_{\mathrm{i}} = \boldsymbol{r}_i + \varepsilon_i \underline{\mathbf{e}}_{1,i} \qquad \textbf{(S1a)}$$

$$\boldsymbol{q}_{\mathrm{i}} = \boldsymbol{r}_i + \varepsilon_i \underline{\mathbf{e}}_{2,i} \qquad \textbf{(S1b)}$$

The lengths of the ***p***- and ***q***-paths are defined as the *eigenvector-following paths* H^* or H:

$$\mathrm{H}^* = \sum_{i=1}^{n-1} |\boldsymbol{p}_{i+1} - \boldsymbol{p}_i| \qquad \textbf{(S1c)}$$

$$\mathrm{H} = \sum_{i=1}^{n-1} |\boldsymbol{q}_{i+1} - \boldsymbol{q}_i| \qquad \textbf{(S1d)}$$

Similar expressions to equations (S**1a-S1b**) and equations (**S1c-S1d**) can be constructed using the stress tensor ellipticity $\varepsilon_\sigma = |\lambda_{2\sigma}|/|\lambda_{1\sigma}| - 1$; note the different numerator and denominator orderings compared with the ellipticity ε. In the limit of vanishing ellipticity ε = 0, *for all* steps *i* along the bond-path then H = BPL. The form of the constituent ***p***- and ***q***-paths along each bond-path (***r***) can be used to provide a 3-D interpretation of bonding to track precisely the mechanisms of bond evolution throughout the functioning of the switch i.e. the hydrogen transfer reaction. Two paths (***q*** *and* ***q′***) are associated with the $\underline{\mathbf{e}}_2$ eigenvector because $\underline{\mathbf{e}}_2 = -\underline{\mathbf{e}}_2$ lie in the same plane for the same point on the bond-path (***r***), correspondingly there are two paths (***p*** and ***p′***) associated with the $\underline{\mathbf{e}}_1$ eigenvector, see **Scheme S1(a)**. The ***q*** (equivalently $\boldsymbol{q}_\sigma$) is always defined to be longer than the ***q′*** (equivalently $\boldsymbol{q}_\sigma'$) because it is constructed from the preferred direction (the $\underline{\mathbf{e}}_2$ eigenvector). Conversely, the ***p*** (equivalently $\boldsymbol{p}_\sigma$) is always defined to be the shorter of the two paths associated with the $\underline{\mathbf{e}}_1$ eigenvector. For very curved bond-paths, however, ***p*** may be shorter than ***r*** (the bond-path length), so we only chose ***p′***; see **Scheme S1(a).**

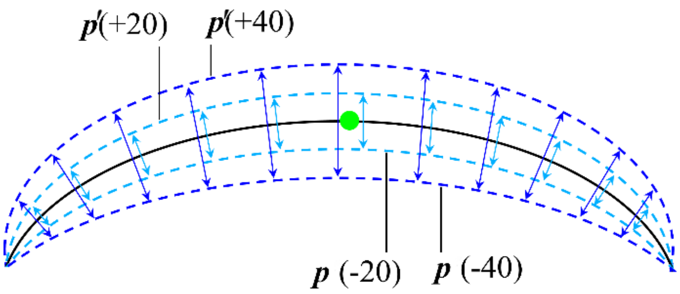

Scheme S1(a). A sketch, not to scale, of the {***p***,***p′***} path-packets illustrating that for the highly curved bond-path (***r***) the ***p***-path may be shorter than ***r***-path.

Implementation details of the calculation of the eigenvector-following path lengths H and H*.

When the QTAIM eigenvectors of the Hessian of the charge density $\rho(\mathbf{r})$ are evaluated at points along the bond-path, this is done by requesting them via a spawned process which runs the selected underlying QTAIM code, which then passes the results back to the analysis code. For some datasets, it occurs that, as this evaluation considers one point after another in sequence along the bond-path, the returned calculated $\underline{\mathbf{e}}_2$ (correspondingly $\underline{\mathbf{e}}_1$ is used to obtain H^*) eigenvectors can experience a 180-degree 'flip' at the 'current' bond-path point compared with those evaluated at both the 'previous' and 'next' bond-path points in the sequence. These 'flipped' $\underline{\mathbf{e}}_2$ (or $\underline{\mathbf{e}}_1$) eigenvectors, caused by the underlying details of the numerical implementation in the code that computed them, are perfectly valid, as these are defined to within a scale factor of -1 (i.e. inversion). The analysis code used in this work detects and re-inverts such temporary 'flips' in the $\underline{\mathbf{e}}_2$ (or $\underline{\mathbf{e}}_1$) eigenvectors to maintain consistency with the calculated $\underline{\mathbf{e}}_2$ (or $\underline{\mathbf{e}}_1$) eigenvectors at neighboring bond-path points, in the evaluation of eigenvector-following path lengths H and H^*, see **Scheme S1(b).**

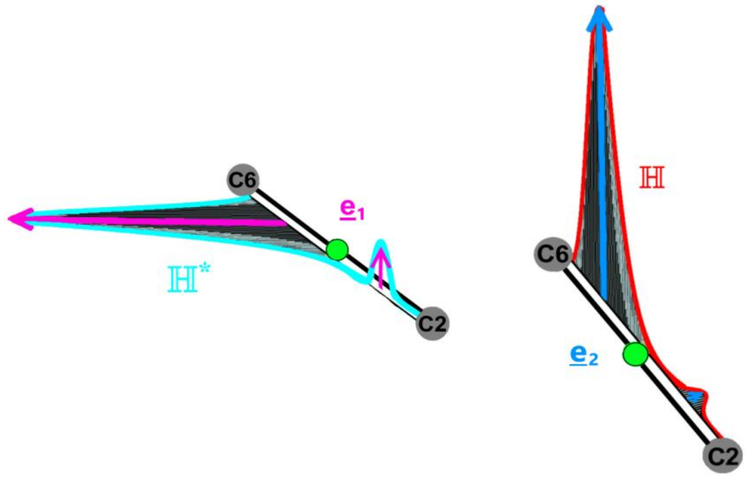

(a) **(b)**

Scheme S1(b). The pale-blue line in sub-figure **(a)** represents the path, referred to as the eigenvector-following path with length H^*, swept out by the tips of the scaled $\underline{\mathbf{e}}_1$ eigenvectors, shown in magenta, and defined by equation **(1c)**. The red path in sub-figure **(b)** corresponds to H, constructed from the path swept out by the tips of the scaled $\underline{\mathbf{e}}_2$ eigenvectors, shown in mid-blue and is defined by equation **(1d)**. The pale-blue and mid-blue arrows representing the $\underline{\mathbf{e}}_1$ and $\underline{\mathbf{e}}_2$ eigenvectors are scaled by the ellipticity ε respectively, where the vertical scales are exaggerated for visualization purposes. The green sphere indicates the position of a given *BCP*.

2. Supplementary Materials S2.

The zeroth order regular approximation (ZORA) to the Dirac equation that includes relativistic corrections can be written as [van Lenthe, E. The ZORA equation. (1996)]; [van Lenthe, E., E.J. Baerends, and J.G. Snijders. Relativistic regular two-component Hamiltonians. *J. Chem. Phys.* **99**, 4597–4610 (1993)]:

$$\left[(\vec{\sigma}\bullet\vec{p})\frac{c^2}{2mc^2-V}(\vec{\sigma}\bullet\vec{p})+V\right]\Psi = i\hbar\frac{\partial\Psi}{\partial t}, \qquad \textbf{(2)}$$

where the term $\vec{\sigma}$ in equation (2) is the Pauli matrix operator, $\vec{p}$ is the momentum operator, c is the speed of light, $c$is the speed of light, m is the mass, V is the potential operator, $i = (-1)^{1/2}$, $\hbar$ is the reduced Plank's constant, Ψ is the wave-function, and t is time. The first term on the left-hand side of equation (2) is the kinetic energy operator. Using the Dirac identity [Dyall, K.G. *Introduction to Relativistic Quantum Chemistry*. (New York: Oxford University Press, 2007], the kinetic operator of the ZORA equation can be decomposed into two parts:

$$(\vec{\sigma}\bullet\vec{p})\frac{c^2}{2mc^2-V}(\vec{\sigma}\bullet\vec{p}) = \vec{p}\bullet\frac{c^2}{2mc^2-V}\vec{p}+i\vec{\sigma}\bullet\left(\vec{p}\left(\frac{c^2}{2mc^2-V}\right)\times\vec{p}\right). \qquad \textbf{(3)}$$

The first operator on the right-hand-side in equation (3) is the scalar relativistic part and the second operator on the right-hand-side is the spin-orbit operator part, which is often neglected. This gives rise to a popular method for including relativity into electronic structure calculations, which is known as the scalar relativistic ZORA (SR-ZORA) equation:

$$\left[\vec{p}\bullet\frac{c^2}{2mc^2-V}\vec{p}+V\right]\Psi = i\hbar\frac{\partial\Psi}{\partial t}. \qquad \textbf{(4)}$$

The applicability of Quantum Theory of Atoms in Molecules (QTAIM) to relativistic case was confirmed by the work of Anderson and Ayers [Anderson, J. S. M. & Ayers, P. W. Quantum Theory of Atoms in Molecules: Results for the SR-ZORA Hamiltonian. *J. Phys. Chem. A* **115**, 13001–13006 (2011)]. They showed that the surface of zero-flux condition required for the Schwinger principle of stationary action for a region [Anderson, J. S. M. & Ayers, P. W. Quantum Theory of

Atoms in Molecules: Results for the SR-ZORA Hamiltonian. *J. Phys. Chem. A* **115**, 13001–13006 (2011)]; [Bader, R. F. W. *Atoms in Molecules: A Quantum Theory*. (Oxford University Press, USA, 1994)] of space as postulated by Bader *et al*. has the same mathematical expression for the SR-ZORA Case. The QTAIM zero-flux condition in the ZORA case can be written as [Anderson, J. S. M. & Ayers, P. W. Quantum Theory of Atoms in Molecules: Results for the SR-ZORA Hamiltonian. *J. Phys. Chem. A* **115**, 13001–13006 (2011)],

$$\nabla\rho^{ZORA}(r) \cdot ds = 0 \qquad \textbf{(5)}$$

where ρ^{ZORA} is the ZORA electron density. Equation **(5)** has the same mathematical expression as in the non-relativistic Bader original formulation but for the ZORA electron density. As in the original Bader formulation, equation **(5)** defines the surfaces, the zero-flux surfaces, of the atomic volumes. The Laplacian of ρ^{ZORA} integrated over any atomic volume Ω vanishes in the ZORA case [Anderson, J. S. M. & Ayers, P. W. Quantum Theory of Atoms in Molecules: Results for the SR-ZORA Hamiltonian. *J. Phys. Chem. A* **115**, 13001–13006 (2011)],

$$\int_{\Omega} \nabla^2 \rho^{ZORA}(r)dr = 0 \qquad \textbf{(6)}$$

The work of Anderson *et al* [Anderson, J. S. M. & Ayers, P. W. Quantum Theory of Atoms in Molecules: Results for the SR-ZORA Hamiltonian. *J. Phys. Chem. A* **115**, 13001–13006 (2011)]. was the first successful of inclusion of relatively into QTAIM. The first computational demonstration of equations(5)-(6) and the ZORA-QTAIM atomic properties and electron density topology for molecules were reported by Anderson *et al*. [Anderson, J. S. M., Rodríguez, J. I., Ayers, P. W. & Götz, A. W. Relativistic (SR-ZORA) quantum theory of atoms in molecules properties. *J. Comput. Chem.* **38**, 81–86 (2017)]. It was shown a ~20-30% (~10-15%) difference between ZORA-QTAIM properties (electron topology) with respect to the non-relativistic ones for gold and other heavy elements [Anderson, J. S. M., Rodríguez, J. I., Ayers, P. W. & Götz, A. W. Relativistic (SR-ZORA) quantum theory of atoms in molecules properties. *J. Comput. Chem.* **38**, 81–86 (2017)]. These results were consistent with the approach of Sadjadi *et al* [Sadjadi, S., Matta, C. F., Lemke, K. H. &

Hamilton, I. P. Relativistic-Consistent Electron Densities of the Coinage Metal Clusters M_2, M_4, M_4^{2-}, and M_4Na_2 (M = Cu, Ag, Au): A QTAIM Study. *J. Phys. Chem. A* **115**, 13024–13035 (2011)].

The reliability of the SR-ZORA for computing properties at *BCP*s was our motivation selecting the method for our investigation [Eickerling, G., Mastalerz, R., Herz, V., Scherer, W., Himmel, H. & Reiher, M. Relativistic Effects on the Topology of the Electron Density. *J. Chem. Theory Comput.* **3**, 2182–2197 (2007)]; [Rodríguez, J. I., Uribe, E. A., Baltazar-Méndez, M. I., Autschbach, J., Castillo-Alvarado, F. L. & Gutiérrez-González, I. Size evolution relativistic DFT-QTAIM study on the gold cluster complexes Au_4-S-C_nH_{2n}-S′-Au_4′ (n=2–5). *Chem. Phys. Lett.* **660**, 287–294 (2016)]. Previous investigations concluded that, at the *BCP*, the SR-ZORA includes most relativistic effects. The 2-component and 4-component methods are usually not required for properties involving the electron density particularly in the case for d-block elements with a high atomic charge, as is the case with gold. They also concluded that if the quantities of interest do not primarily rely on spin-orbit interactions, the error change at the *BCP* tends to be negligible. The SR-ZORA formalism has also been used for reliable investigations at the *BCP* in several computational studies. [Eickerling, G., Mastalerz, R., Herz, V., Scherer, W., Himmel, H. & Reiher, M. Relativistic Effects on the Topology of the Electron Density. *J. Chem. Theory Comput.* **3**, 2182–2197 (2007)].

3. Supplementary Materials S3.

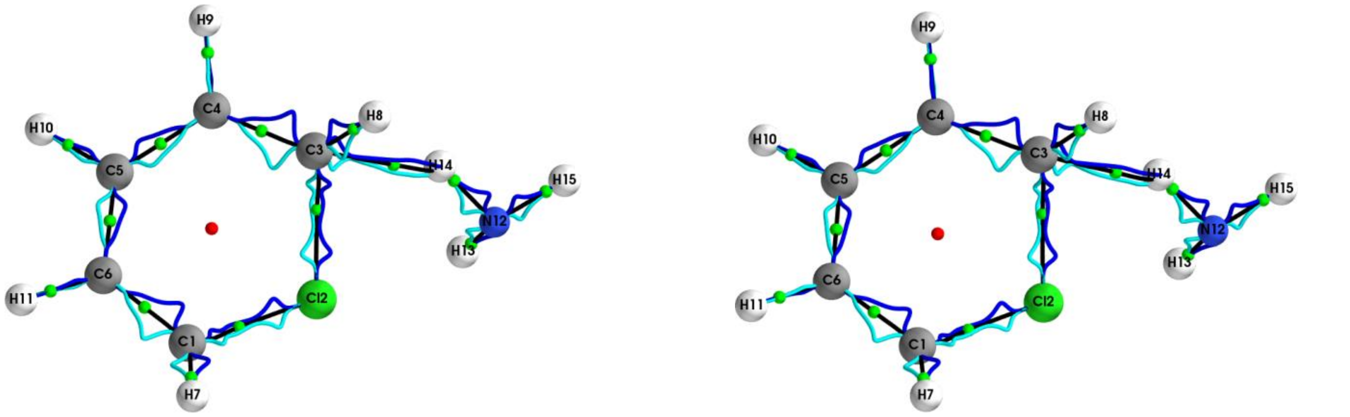

(a)

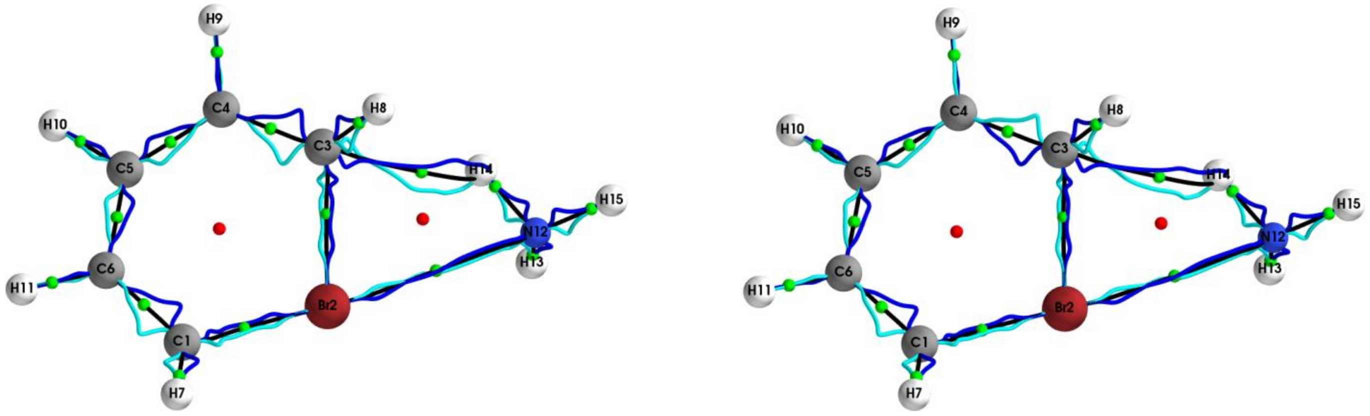

(b)

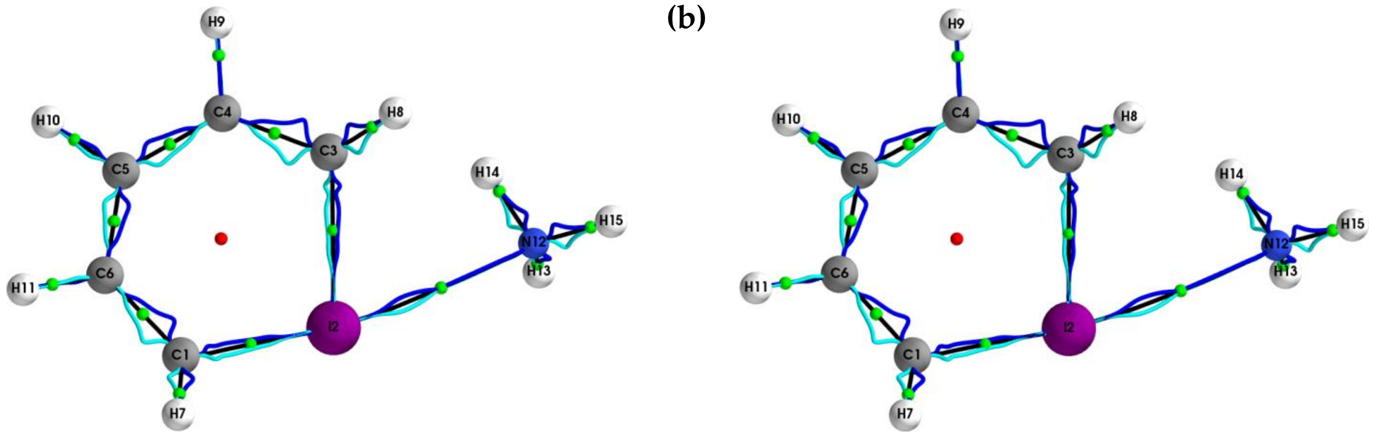

(c)

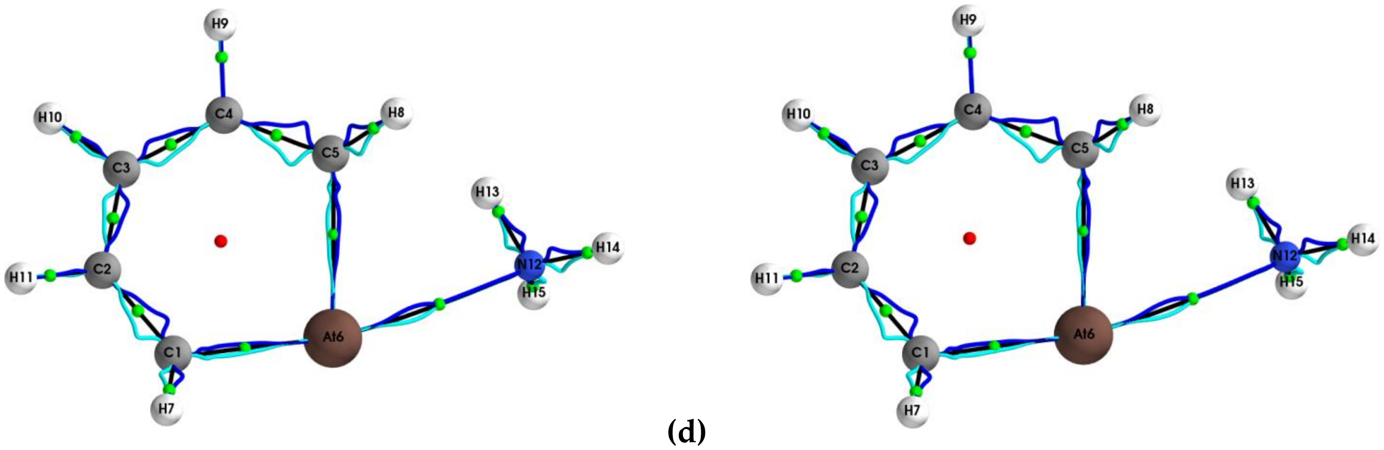

(d)

Figure S3 The {***p,p′***} path-packets, ***p***(dark-blue) and ***p***'(light-blue) for the (Y = Cl, Br, I, At): halogenabenzene: NH_3 system calculated with ECPs are presented in sub-figure **(a-d)** respectively, see the caption of **Figure 1** for further details

Table S4. The ECP results for the (H,H′), (H^*,$H^{*\prime}$), (BPL, GBL_II), (H_σ,H_σ'), (H_σ^*,$H_\sigma^{*\prime}$), ξ($\mathbf{r}_b$), S and $H(\mathbf{r}_b)$ of the (Y = Cl, Br, I, At): 1-methyluracil: NH_3 system at the ΔE_{min}. Note the use of the bond notations "--" (closed-shell *BCP*) and "-" (shared-shell *BCP*).

BCP	(H,H′)	(H^*,$H^{*\prime}$)	(BPL, GBL)	(H_σ,H_σ')	(H_σ^*,$H_\sigma^{*\prime}$)	ξ($\mathbf{r}_b$)	S	$H(\mathbf{r}_b)$
**Cl-NH$_3$**								
C3--H14	(5.377, 5.208)	(5.257, 5.285)	(4.922, 4.864)	(5.011, 4.928)	(4.958, 4.973)	0.339	0.155	0.001
N12-H14	(2.166, 2.158)	(2.162, 2.162)	(1.871, 1.914)	(1.892, 1.892)	(1.892, 1.892)	-0.188	1.635	-0.523
C3-H8	(2.365, 2.343)	(2.340, 2.369)	(2.003, 2.036)	(2.055, 2.040)	(2.043, 2.051)	-0.246	1.740	-0.342
C4-C3	(3.198, 3.185)	(3.173, 3.212)	(2.587, 2.585)	(2.657, 2.639)	(2.644, 2.651)	-0.285	3.036	-0.418
Cl2-C3	(3.996, 3.935)	(3.949, 3.969)	(3.427, 3.396)	(4.060, 4.059)	(3.924, 3.967)	-0.745	0.855	-0.131
**Br-NH$_3$**								
C3--H14	(6.255, 5.574)	(5.799, 5.855)	(5.123, 4.991)	(5.331, 5.105)	(5.202, 5.214)	0.324	0.112	0.001
Br2--N12	(6.426, 6.410)	(6.373, 6.456)	(6.107, 6.091)	(6.144, 6.126)	(6.120, 6.143)	0.274	0.102	0.002
N12-H14	(2.166, 2.157)	(2.161, 2.162)	(1.871, 1.913)	(1.891, 1.891)	(1.891, 1.891)	-0.188	1.637	-0.524
C3-H8	(2.331, 2.310)	(2.306, 2.336)	(2.005, 2.037)	(2.053, 2.038)	(2.041, 2.049)	-0.247	1.747	-0.341
C4-C3	(3.144, 3.134)	(3.119, 3.161)	(2.587, 2.585)	(2.653, 2.634)	(2.641, 2.646)	-0.286	3.027	-0.417
Br2-C3	(3.983, 3.934)	(3.952, 3.956)	(3.696, 3.666)	(3.724, 3.722)	(3.710, 3.736)	-1.022	0.738	-0.089
**I-NH$_3$**								
I2--N12	(6.071, 6.056)	(6.062, 6.064)	(5.983, 5.970)	(6.639, 6.638)	(6.464, 6.665)	0.339	0.150	0.001
C3-H8	(2.231, 2.214)	(2.211, 2.234)	(2.008, 2.039)	(2.041, 2.031)	(2.032, 2.039)	-0.253	1.692	-0.334
C4-C3	(2.977, 2.973)	(2.956, 2.995)	(2.589, 2.587)	(2.637, 2.621)	(2.628, 2.630)	-0.297	2.679	-0.405
I2-C3	(4.160, 4.129)	(4.143, 4.143)	(4.024, 4.004)	(4.687, 4.685)	(4.513, 4.715)	-1.845	0.623	-0.064
**At-NH$_3$**								
At6--N12	(6.006, 5.989)	(5.996, 5.998)	(5.925, 5.914)	(7.260, 7.259)	(6.797, 7.426)	0.342	0.156	0.001
C5-H8	(2.251, 2.231)	(2.230, 2.252)	(2.012, 2.043)	(2.048, 2.037)	(2.038, 2.046)	-0.254	1.687	-0.331

C4-C5	(2.951, 2.946)	(2.928, 2.970)	(2.593, 2.591)	(2.641, 2.623)	(2.631, 2.633)	-0.298	2.650	-0.402
At6-C5	(4.331, 4.307)	(4.318, 4.319)	(4.235, 4.208)	(5.565, 5.563)	(5.125, 5.741)	-22.35	0.483	-0.047
